# DermACELL: a novel and biocompatible acellular dermal matrix in tissue expander and implant-based breast reconstruction

**DOI:** 10.1007/s00238-014-0995-8

**Published:** 2014-07-31

**Authors:** Jamal M. Bullocks

**Affiliations:** Plastic Surgery Division, Baylor College of Medicine, One Baylor Plaza, Houston, TX 77030 USA

**Keywords:** Breast, Acellular dermal matrix, Two-stage reconstruction, Tissue expanders

## Abstract

**Background:**

Acellular dermal matrices present a new alternative to supporting expanders and implants for breast reconstruction in breast cancer patients following mastectomy. However, some studies have suggested that acellular dermal matrix may increase the complication rates in certain clinical settings. DermACELL acellular dermal matrix offers advanced processing in order to attempt to decrease bio-intolerance and complications.

**Methods:**

Ten consecutive patients that presented for breast reconstruction and were candidates for tissue expanders underwent the procedure with the use of an acellular dermal matrix. The patients underwent postoperative expansion/adjuvant cancer therapy, then tissue expander exchange for permanent silicone breast prostheses. Patients were followed through the postoperative course to assess complication outcomes. Histologic evaluation of host integration into the dermal matrix was also assessed.

**Results:**

Of the ten patients, eight completed reconstruction while two patients failed reconstruction. The failures were related to chronic seromas and infection. Histology analysis confirms rapid integration of mesenchymal cells into the matrix compared to other acellular dermal matrices.

**Conclusions:**

Based on our observations, DermACELL is an appropriate adjunct to reconstruction with expanders. Histological analysis of vascularization and recellularization support the ready incorporation of DermACELL into host tissue.

Level of Evidence: Level IV, therapeutic study.

## Introduction

Following mastectomy, tissue expander reconstruction is one of the most common methods for breast reconstruction [1]. The use of acellular dermal matrices (ADMs) has been popularized in the use of tissue expander reconstruction. This biologic matrix provides support in the lower pole of the breast and helps define the lower pole and inframammary fold. It also speeds the rate of expansion by reducing tension of the mastectomy skin and allows higher initial fill volumes. These two attributes help to decrease the overall length of the reconstruction process. Most recently, it has been suggested that ADMs help prevent capsular contracture, a common long-term problem with implants in the reconstructed breast [2–4]. Although our institutional experience has been promising, other studies have suggested mixed, although generally positive, findings concerning the use of ADMs [3, 5]. Since clinical procedures are generally standardized, this suggests that the relative ability of the various ADMs to incorporate into soft tissues may relate to the manufacturing or processing of the particular material. Incorporation confers biocompatibility, which ultimately creates symmetry within the host. Bio-incompatibility due to lack of incorporation may cause seroma, infection, and reconstructive failure [6]. The reconstructive process can be difficult enough for the patient without adding further cosmetic complications. Structural support of the breast is necessary to provide the ideal breast shape and implant position. ADMs have been recognized as being beneficial by serving as a supplemental graft in breast reconstruction cases. They can supplement muscle coverage, shape the breast and inframammary fold, and possibly reduce the number of surgeries patients need to undergo. This technique utilized in breast reconstruction brings with it many benefits [7].

Acellular dermal matrices are composed of the dermal layer and extracellular matrix of thin layers of donated skin that have had the epidermal layer removed. Donor cellular material including major histocompatibility complex (MHC) proteins are removed in a series of further treatments to theoretically minimize immunological response in ADM recipients [8]. The ability of ADMs to promote significant revascularization and cellular infiltration make them an encouraging option for an array of tissue regeneration applications, including wound healing, soft tissue reconstruction, and augmentation [9–27].

A recently developed human ADM produced by an anionic detergent and endonuclease-based decellularization process [28] is a product trade-named DermACELL® (LifeNet Health, Virginia Beach, VA, USA). Unique features of this allograft include removal of at least 97 % DNA, an indication of complete decellularization and potentially reduced immunogenicity, terminally sterilization to a Sterility Assurance Level of 10^−6^, and being provided at room temperature and ready to use directly out of the package.

Here, we examine the post-mastectomy outcome of a two-stage breast reconstruction using this new human acellular dermal matrix, DermACELL, in a 10 consecutive patient prospective cohort series.

## Patients and methods

### Case series overview

This prospective, consecutive cohort series included 10 female patients ranging in age from 33 to 59 years old who underwent mastectomies and presented to have breast reconstructions with the use of DermACELL. Of the 10 patients, eight had bilateral mastectomies and two had unilateral mastectomies, for a total of 18 breasts. One patient elected to have a prophylactic bilateral mastectomy after testing positive for the BRCA-1 gene. Two patients underwent unilateral mastectomies, one of whom had previously undergone a unilateral mastectomy and reconstruction of the contralateral breast. The remaining patients each presented with breast cancer unilaterally and elected to have a mastectomy of the affected breast, as well as prophylactic mastectomy of the contralateral breast. Of the 10 patients, two were smokers (Table 1). Two patients required neo-adjuvant chemotherapy, one required adjuvant chemotherapy, one additional patient required both neo-adjuvant and adjuvant chemotherapy, and two required radiation.

**Table 1:** Patient overview

Patient no.	Age (years)	Post-op chemotherapy	Radiation	Uni- or bilateral	Duration of implant prior to 2nd stage (weeks)	Expansion volume	Smoker	Implant volume
1	50	Adj.	No	Bilateral	46	400 cc	No	400 cc
2	43	No	No	Bilateral	24	400 cc	No	600 cc
3	52	No	No	Bilateral	9	380 cc	No	425 cc
4	43	Neo-adj./Adj.	Yes	Bilateral	N/A	X	Yes	X
5	33	No	No	Bilateral	7	550 cc	No	500 cc
6	56	Neo-adj.	No	Bilateral	N/A	X	Yes	X
7	59	No	No	Bilateral	20	700 cc	No	700 cc
8	45	Neo-adj.	Yes	Unilateral (R)	41	600 cc	No	Flap^a^
9	48	No	No	Unilateral (R)	43	220 cc	No	175 cc
10	33	No	No	Bilateral	20	500 cc	No	555 cc

^a^Planned autologous reconstruction in a delayed fashion

Breast reconstruction was performed in two stages. In the first stage, DermACELL and expanders were used. Following tissue expansion, patients advanced to the second stage of the procedure of immediate reconstruction for final placement of silicone implants.

### Clinical procedures

Each reconstruction included placement of Mentor CPX3 tall height contour profile implants (Mentor Worldwide LLC, Santa Barbara, CA, USA) for tissue expansion and 16 × 6 cm^2^ DermACELL patches, with one patient’s DermACELL patch tailored down to 12 × 6 cm^2^. All expanders were placed with an initial volume of 100 cc intraoperatively using sterile saline. The pectoris muscle was elevated and the DermACELL was sutured to the inferior portion of the muscle and to the inframammary fold to serve as a sling. A drain was placed in the breast pocket and in the subcutaneous pocket between the mastectomy skin and the DermACELL. Two weeks postoperative expansion began and continued between 7 and 46 weeks (average 8 weeks) until the desired volume. Drains were removed between 6 and 21 days postoperatively (average 15 days) depending on their output.

### Histological analysis

Biopsy samples were taken from the patients and sent in formalin to Dominion Pathology Laboratories (Norfolk, VA, USA) for sectioning and staining. Hematoxylin and eosin (H&E) staining was undertaken to assess cellularity and general ultrastructure. Additionally, immunohistochemical staining for the endothelial cell marker CD34 (CD34) was used to assess vascularity and Verhoef-Von Gieson (VVG) staining was performed to assess elastic fibers and general ultrastructure. Histological assessments were determined by dermatopathologists Kevagn Fair, MD (Dominion Pathology) and Antoinette Hood, MD (Eastern Virginia Medical School, Norfolk, VA, USA).

## Results

### Patient overview and results

Eight (15 breasts) of the 10 breast reconstruction procedures were successfully completed and only minor complications were reported in two patients (Table 2). Major complications were seen in the two patients with failed breast reconstructions due to smoking and infection. Patient outcomes were as follows (Table 2): four breasts developed seromas, there were two surgical site infections, four experienced delayed healing, and three had flap necrosis. Of particular note, there was no observation of hematomas or red breast syndrome in any patient. Over half of the observed complications were limited to a single patient, limiting the complicative profile and associating patient comorbidities as the major contributor. It appears that a history of smoking caused mastectomy skin necrosis and a port-a-cath wound infection contributed to the failed reconstruction. This patient developed seromas of both breasts, a purulent right breast infection, and mastectomy flap necrosis of both breasts. This patient also had postoperative radiation and significant skin necrosis, apparently due to the radiation. She also eventually developed metastatic cancer and did not return to undergo reconstruction. The other patient who failed reconstruction developed a seroma, surgical site infection, flap necrosis, and experienced delayed healing of the left breast. These complications were most likely the result of the patient continuing to smoke throughout her postoperative recovery. The expanders were removed and the reconstruction was aborted.

**Table 2:** Patient outcomes

Patient no.	Seroma	Hematoma	Surgical site infection	Red breast	Delayed healing	Flap necrosis	Completed reconstruction	Failed reconstruction
1	0/2	0/2	0/2	0/2	0/2	0/2	2/2	0/2
2	0/2	0/2	0/2	0/2	0/2	0/2	2/2	0/2
3	0/2	0/2	0/2	0/2	0/2	0/2	2/2	0/2
4	2/2	0/2	1/2	0/2	2/2	2/2	0/2	2/2
5	1/2	0/2	0/2	0/2	0/2	0/2	2/2	0/2
6	1/2	0/2	1/2	0/2	1/2	1/2	0/2	2/2
7	0/2	0/2	0/2	0/2	0/2	0/2	2/2	0/2
8	0/1	0/1	0/1	0/1	0/1	0/1	1/1	0/1
9	0/1	0/1	0/1	0/1	1/1	0/1	1/1	0/1
10	0/2	0/2	0/2	0/2	0/2	0/2	2/2	0/2
Total	4/18	0/18	2/18	0/18	4/18	3/18	15/18	4/18

**Fig. 1:**
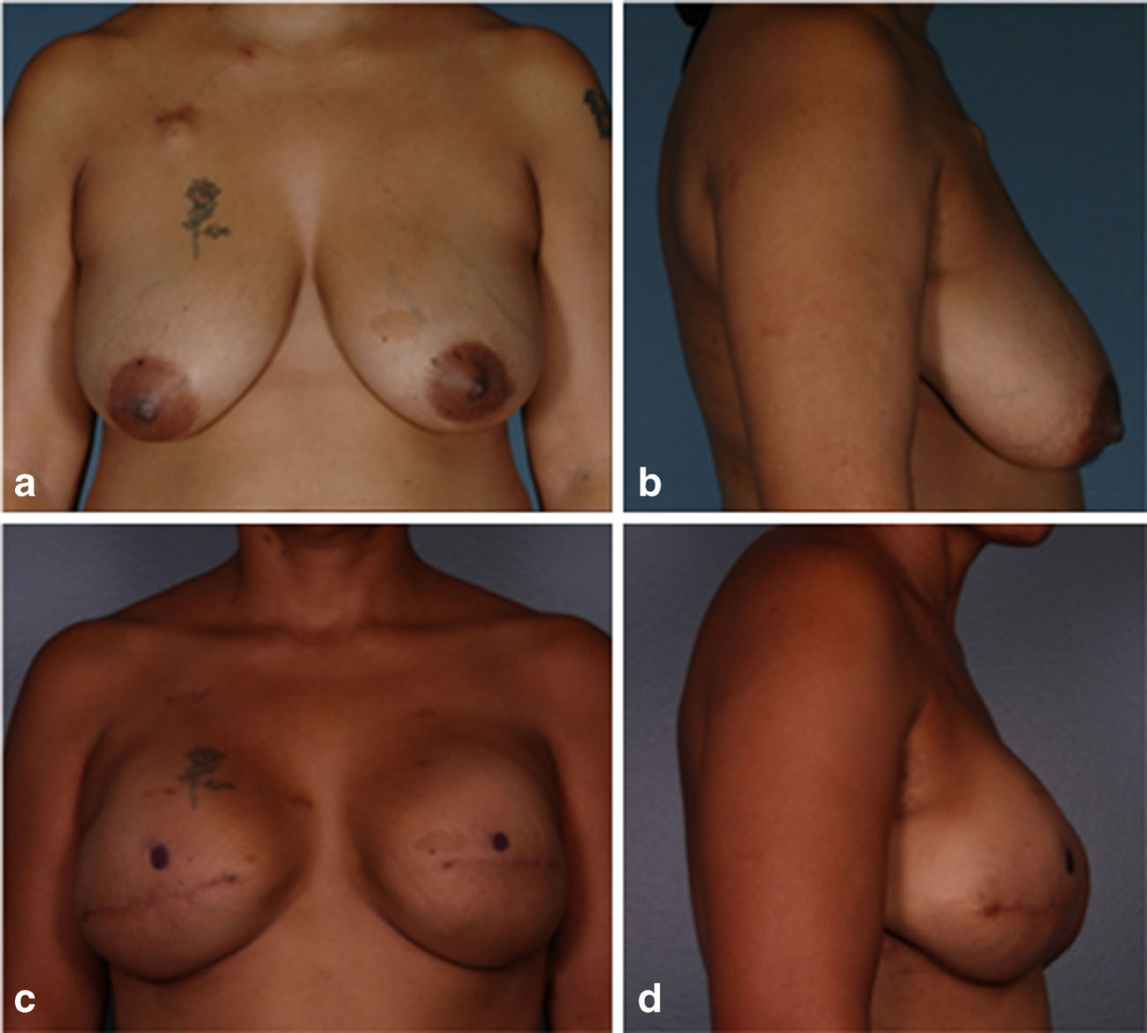
**a**, **b** Patient 2 preoperative before undergoing bilateral mastectomy with immediate reconstruction. **c**, **d** Patient 2 postoperative 7 months after placement of permanent implant

**Fig. 2:**
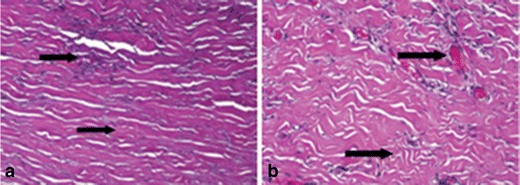
**a**, **b** H&E stain of DermACELL sample taken from patient 2’s left breast at 7 months postoperative. Viewed under ×100. The *upper arrows* identify blood vessels while the *lower arrows* identify fibroblasts

Six patients that completed reconstruction did not experience any complications. Patient 2 (Fig. 1) was one of these six patients that had a successful reconstruction with no infections, seromas, or other possible complications. She was 43 years old, had neoadjuvant chemotherapy for a left locally advanced breast cancer, and was advised to have a mastectomy. The patient also requested a contralateral prophylactic mastectomy with immediate reconstruction of both breasts. From histological analysis (Fig. 2), it is evident that the DermACELL had incorporated into the host tissue and allowed for revascularization. The tissue was infiltrated with fibroblasts and blood vessels.

**Fig. 3:**
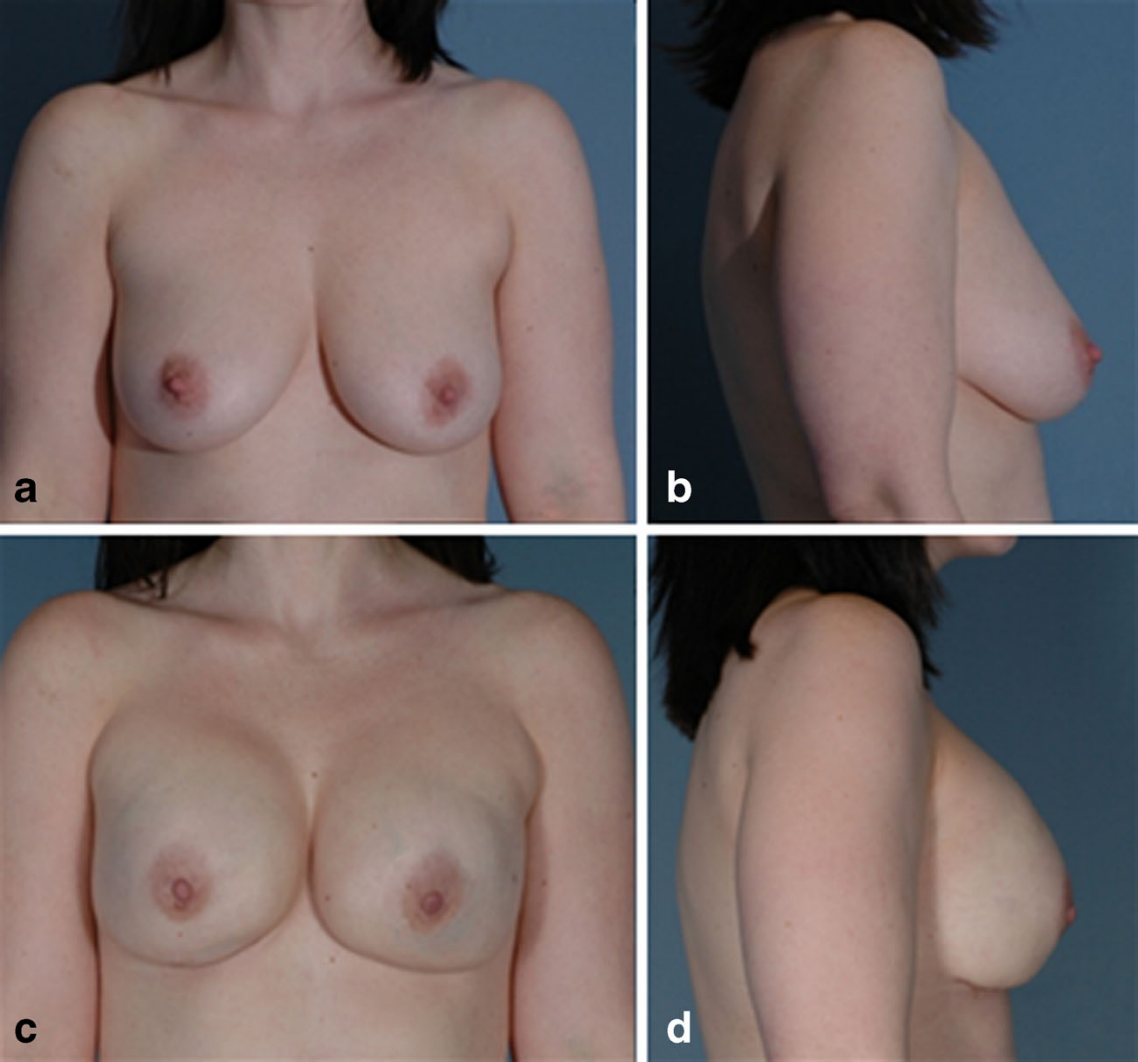
**a** Patient 5 preoperative before undergoing bilateral mastectomy with immediate reconstruction. **b** Patient 5 postoperative 1 month after exchange of tissue expander for implant

Another notable completed reconstruction was performed on patient 5 (Fig. 3). She was 33 years old, had a strong family history of breast cancer, and therefore decided to be tested for the BRCA-1 gene mutation. The patient tested positive for the mutation and elected to undergo prophylactic mastectomy of both breasts with immediate breast reconstruction. A seroma developed on the left breast between the skin and the DermACELL on day 10. A drain was placed between the skin and DermACELL, and was then removed 1 week later. The patient had expansion for about 7 weeks and then returned for tissue expander removal and placement of the permanent implants. At this time, the DermACELL was found to be incorporated into the surrounding tissues.

**Fig. 4:**
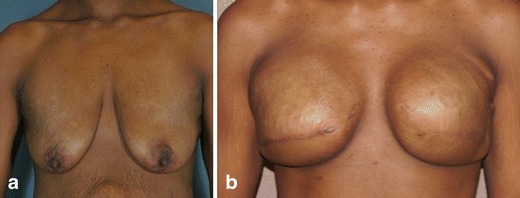
**a** Patient 10 preoperative before undergoing bilateral mastectomy with immediate reconstruction. **b** Patient 10 postoperative 7 months after mastectomy and placement of tissue expanders and DermACELL

**Fig. 5:**
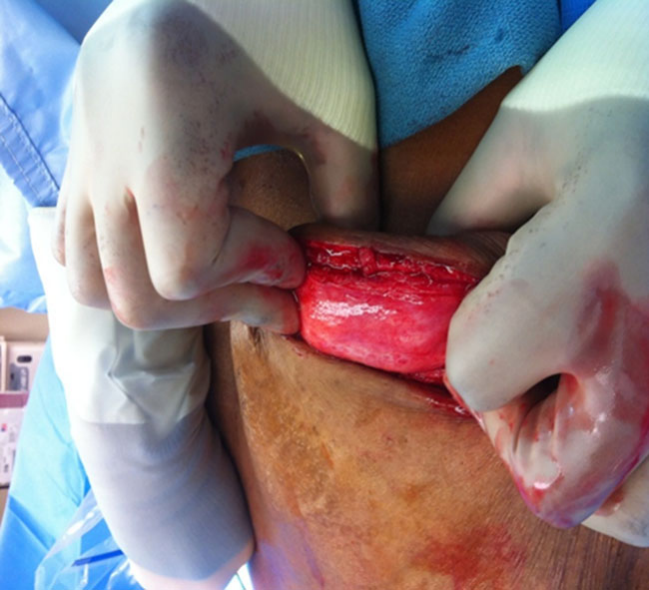
Patient 10, 5 months after expansion of the DermACELL, the matrix is incorporated into the surrounding tissue and show signs of granulation

Patient 10, one of the eight patients with completed reconstruction, was a 33-year old who originally presented for breast augmentation (Fig. 4). During her preoperative screening, she was found to have left breast cancer and was counseled to undergo mastectomy. The patient agreed to have left breast mastectomy and also opted to have prophylactic right breast mastectomy followed by immediate breast reconstruction. After expansion continued to the desired volume, the tissue expander implants were removed at 20 weeks and replaced with permanent silicone breast implants. During placement of the permanent implants, it was noted that the DermACELL matrix had incorporated into the surrounding tissue and showed signs of granulation budding (Fig. 5).

### Histological analysis

**Fig. 6:**
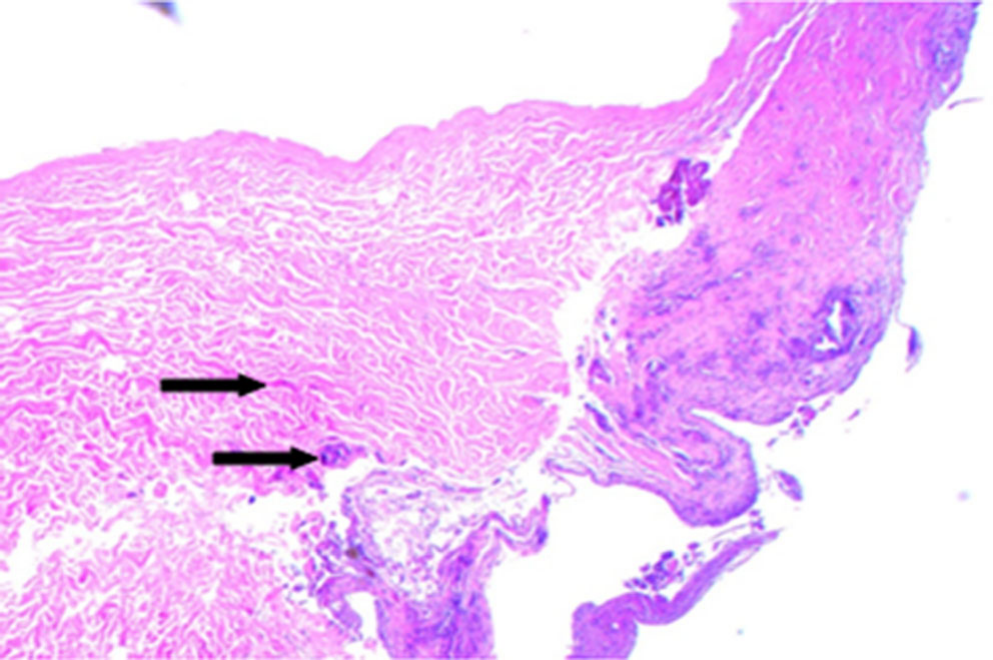
Hematoxylin and eosin staining of biopsy from patient #1 following 16 weeks in situ placement of acellular dermal matrix. Note the intact ultrastructure and also evidence of cellular in-growth as apparent fibroblasts (*arrows*) at ×10 magnification

**Fig. 7:**
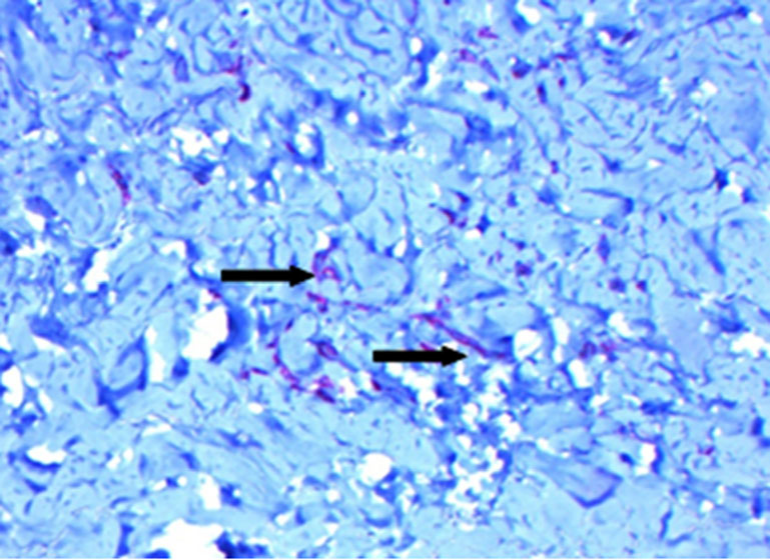
CD34 staining of biopsy from patient #1 following 16 weeks in situ placement of acellular dermal matrix. Evidence of robust vascularization is noted by reddish-brown stains apparently associated with blood vessels (*arrows*) at ×10 magnification

**Fig. 8:**
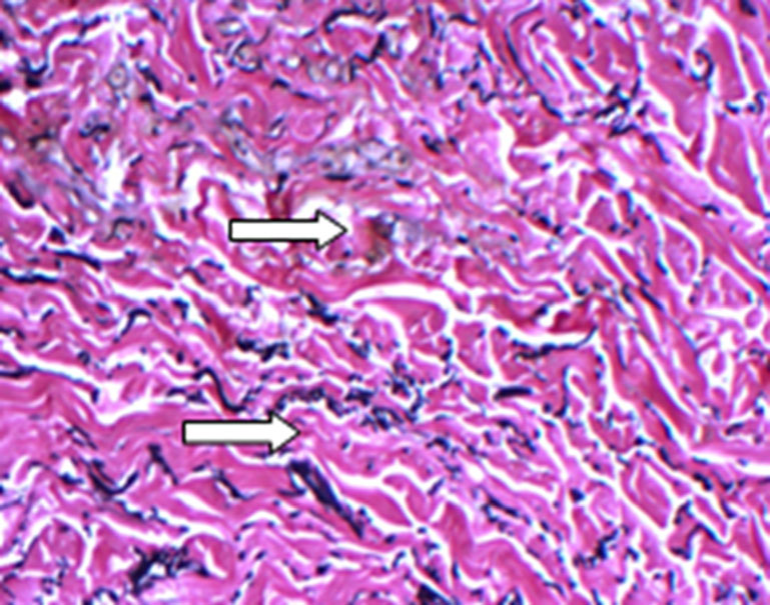
Verhoef-VanGeisen staining of biopsy from patient #1 following 16 weeks in situ placement of acellular dermal matrix. Note abundance of elastin (*arrows*) in this ×10 magnification

Histology analysis confirms the incorporation of the DermACELL matrix into the surrounding tissue, aiding in reconstruction. Observations for all biopsy samples included presence of fibroblasts, intact ultrastructure including elastin, and vasculature (Figs. 6, 7, and 8). Rarely, a foreign body response was observed and was noticeable at the same position as polarizable material. The foreign body response was consistent with suture material, as evidenced by the regular pattern and minimum associated inflammation. Pseudo-capsule formation was generally seen on the side of the implant facing the expander, which occurred as a benign response to the expander material. The opposite interface between the implant and the host tissue exhibited some tissue integration and minimal inflammation, which was consistent with normal healing. The implant material looked more organized with less vasculature and fewer living cells compared to host tissue. These findings are consistent for a stable material being slowly incorporated and remodeled after a few weeks to a few months after surgery when biopsy samples were taken.

## Discussion

Overall, excellent results were noted with DermACELL in two-stage breast reconstruction. This consecutive cohort series did not exclude challenging patients. Most complications were isolated to two compromised patients. The most complications were observed in the patient who was an occasional smoker. This was not unexpected as Anthony et al. [29] noted a trend towards significance in their study on the incidence of complications in breast reconstruction using acellular dermal matrices. Padubidri et al. [30] demonstrated a significant complication rate for smokers in their retrospective study of hundreds of breast reconstruction patients. Other studies have also noted an increased incidence of complications of patients who smoke after post-mastectomy breast reconstructions [31, 32].

In addition to the immediate advantages of providing structural support, the ultimate goal of using ADM’s is to enable integration of the matrix into the host tissue. This integration can be quantified by examining the revascularization and recellularization of the ADM. The two blinded dermatopathologists, who assessed DermACELL samples, identified numbers of blood vessels and fibroblasts that support incorporation into the host cell.

A similar case series [33] was recently published where DermACELL was used for two-stage breast reconstructions in 10 breast cancer patients. After excluding one patient for tobacco use, the author found comparable results with 14 out of 17 breasts successfully reconstructed to the second stage implant sequence. There were few complications reported, including the noteworthy absence of red breast syndrome in all patients, and most postoperative complications occurred in a patient who relapsed into tobacco use. Histological analysis of biopsy samples taken of the ADM from eight patients indicated incorporation with minimal inflammation and normal healing, consistent with our findings.

Another study [34] using in vivo rat models compared several different ADM’s, including DermACELL. The results of the study of Capito et al. support our findings. At day 7 postoperative, the DermACELL matrices had nearly twice the number of blood vessels as the next highest ADM. In regards to recellularization, it was found that DermACELL had the statistically significant highest amount of cell density at days 7, 14, and 21 postoperative as well as the greatest amount of cell infiltration.

The success reported here with DermACELL may be due to the processing and sterility of the material. The process involves the use of a solution of non-denaturing anionic detergent (N-Laurol sarcosinate), recombinant endonuclease (Benzonase® (Merck KGaA, Darmstadt, Germany), and antibiotics (Polymixin B, Vancomycin, and Lincomycin), resulting in a material that exhibits at least 97 % nucleic acid removal, while maintaining biomechanical strength. DermACELL allografts are preserved in glycerol, stored at room temperature, and are ready to use without needing to be rehydrated [35]. The allografts are terminally sterilized at ultra-low temperatures to achieve a 10^−6^ sterility assurance level [35, 36]. The temperature of irradiation has proved to be important because sterilizing grafts at very low temperatures minimizes free radicals and eventual tissue damage [37, 38]. In addition to the temperature at which irradiation occurs, the solutions used to clean the human tissue can determine the quality of the allograft. When harsh chemicals such as acetone, sodium hydroxide, and hydrogen peroxide are used, they can greatly damage the soft tissue [39]. Many factors are involved in the processing of a human tissue allograft with a sterility assurance level of 10^−6^. An allograft with a sterility assurance level of 10^−6^ is equivalent to the level of sterility provided with implantable medical devices, and the use of such sterile allografts could result in a lower rate of infection than when using autografts obtained from a second procedural site [40, 41]. By using low doses of gamma irradiation at very low temperatures, this process is able to ensure a high degree of safety without compromising biological and biomechanical properties [42–46]. The decellularization process used to prepare DermACELL is designed to remove cellular elements while retaining matrix structure, theoretically yielding a material with reduced antigenicity, increased biocompatibility, and subsequently reduced complications.

AlloDerm® (LifeCell Corporation, Bridgewater, NJ, USA), a decellularized human dermal matrix, is aseptically processed and freeze-dried. It is treated with a buffered salt solution to separate and eliminate the epidermis, then washed with a series of mild non-denaturing detergent solutions to solubilize and eliminate all cells [47]. AlloDerm undergoes a sterilization process that includes electron beam irradiation, which provides for a sterility assurance level of 10^−3^. FlexHD® (Musculoskeletal Transplant Foundation, Edison, New Jersey, USA) is human allograft skin that is minimally processed to remove epidermal and dermal cells, a process that maintains the integrity of the matrix. It is packaged in an ethanol solution and must be soaked in a sterile solution before implantation. The allograft tissue complies with the requirements of United States Pharmacopeia (USP) <71> Sterility tests but is not terminally sterilized, and thus claims no level of sterility assurance [48].

While all of these products are acellular dermal matrices, there are many differences between the three, including the degree of sterilization. DermACELL maintains a sterility assurance level of 10^−6^, which assures that no more than one in a million implants could potentially be infected with microorganisms. In comparison, AlloDerm is only aseptic with a sterility assurance level of 10^−3^ and, in theory, about one in one thousand implants could potentially be infected. FlexHD is not terminally sterilized, but meets the requirements of USP <71> Sterility tests.

The same sterility standards are not followed by all organizations that supply bone and tissue allografts. Since there is no standard definition of sterile, there can be clinical concern and confusion regarding infection [49]. Contaminated allografts can cause significant complications or even death in patients who receive the infected tissue [50]. In addition to compromising the health of patients, surgical site infections due to contaminated allografts can place a notable financial burden on patients, hospitals, and health care providers. As determined by the Centers for Medicare and Medicaid Services (CMS), surgical site infections are considered to be preventable complications and hospitals will not be reimbursed for the care of patients suffering from such complications [51]. In 2005, a study performed in Pennsylvania reported that the average charge for patients that developed a hospital infection ($173,206) was about four times as high as the charge for patients with the same diagnosis who did not contract an infection ($44,367) [52]. Therefore, allograft-associated infections place additional financial burden on patients and have a significant economic impact on hospitals. Rigorous screening, in combination with stringent aseptic tissue processing followed by terminal sterilization, can help eliminate allograft-associated infections [48].

Decellularization of human allograft tissue performed during tissue processing results in a material devoid of immunogenic components, theoretically allowing for improved graft biocompatibility, incorporation, and healing. A key measure of the effectiveness of decellularization is removal of DNA. The decellularization process used in the treatment of DermACELL results in at least a 97 % reduction in the DNA content of the tissue, reduced, in one analysis, to approximately 15.97 ng/mg dry weight. The residual DNA value for AlloDerm is much higher at 272.8 ng/mg dry weight [36].

The presence of erythema of mastectomy skin flaps following matrix implant, also known as “red breast syndrome,” has been reported when using either human or xenograft matrices [20, 53, 54]. While not fully understood, it is suggested that this may be an inflammatory response to the implanted matrix or preservatives [20, 53]. It is of special interest to note that there were no cases of red breast syndrome in this case series using DermACELL. While no generalizable conclusions can be reached from this small patient population, the processing of DermACELL ADM resulting in significant reduction in DNA and cellular content may result in the absence of erythema.

**Fig. 9:**
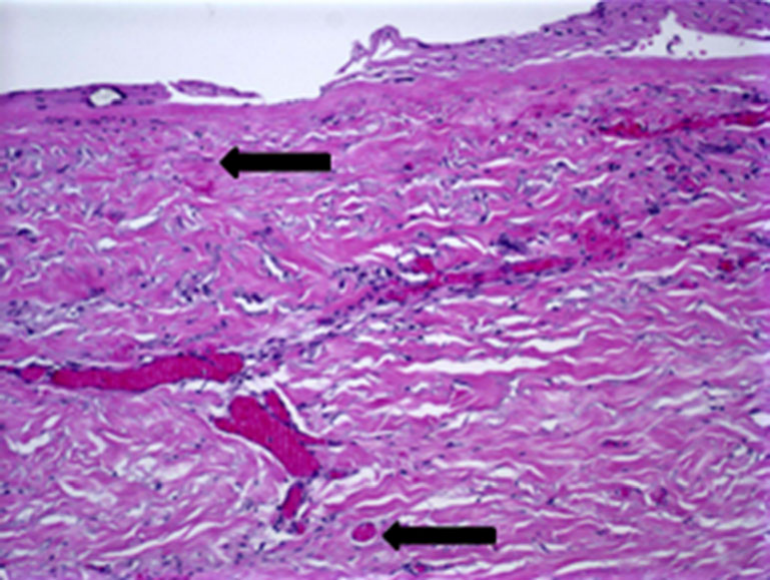
Six-week postoperative biopsy for DermACELL. The *upper arrow* identifies a fibroblast and the *lower arrow* identifies a blood vessel

**Fig. 10:**
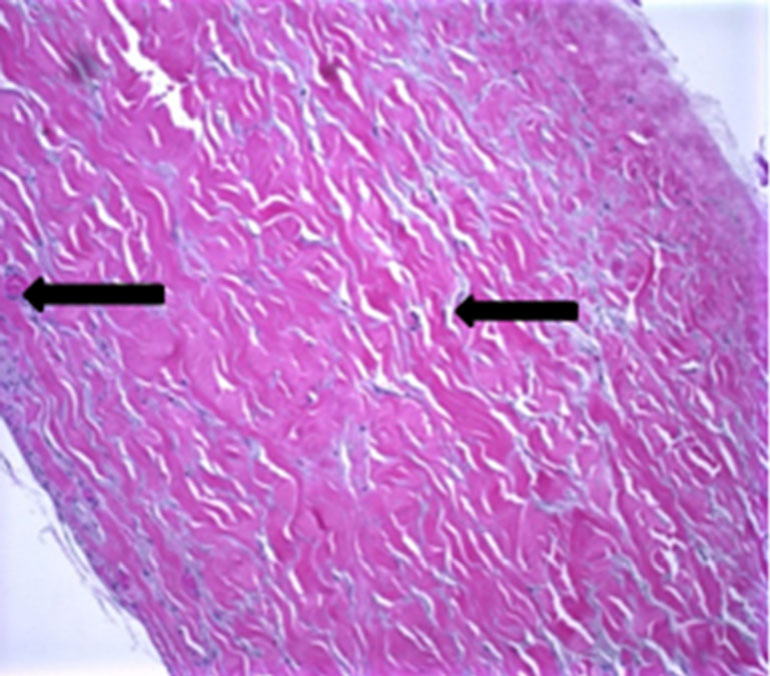
Postoperative biopsy for AlloDerm. The *arrow on the left* identifies a blood vessel and the *arrow on the right* identifies a fibroblast

**Fig. 11:**
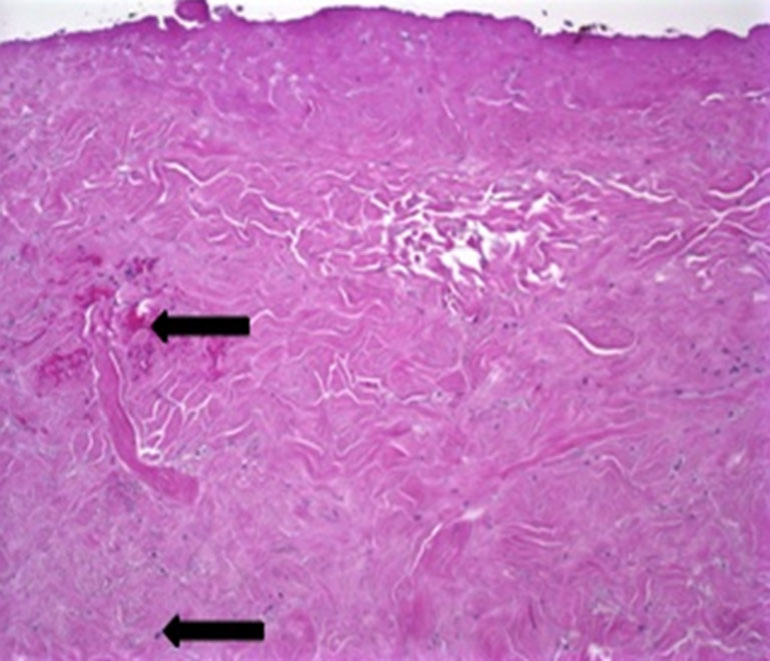
Postoperative biopsy for FlexHD. The *upper arrow* identifies a hematoma and the *lower arrow* identifies a fibroblast

To compare the effectiveness of different acellular dermal matrices, histological analysis was performed on biopsies taken as part of this cohort and from additional two-stage breast reconstruction patients, also treated by this study author. One patient had received AlloDerm, while FlexHD was used in the second patient. At 6 weeks postoperative, DermACELL tissue was infiltrated with fibroblasts and blood vessels (Fig. 9). The revascularization of the DermACELL tissue indicates that incorporation has occurred. In comparison, the AlloDerm tissue biopsy (Fig. 10) did not have nearly as many blood vessels and fibroblasts as the DermACELL tissue. A postoperative biopsy of FlexHD also showed a lack of revascularization (Fig. 11) in our study at the same time point. Further studies are needed for direct comparisons between DermACELL and other ADMs.

It is important for successful breast reconstructions utilizing acellular dermal matrices to facilitate the incorporation of the host tissue into the allograft material. Acellular dermal matrices are able to support larger and faster tissue expansion, reduce capsular contracture, and decrease the rate of revision [55–58]. The ability of DermACELL to incorporate into host tissue with good revascularization and recellularization could provide for quicker breast reconstructions with fewer complications.

## Conclusion

DermACELL appears to be an appropriate adjunct to reconstruction with expanders. Overall, patients that experienced the most complications postoperatively were those with unhealthy lifestyle behaviors, most notably smoking. The results of our case series suggest that DermACELL is at least comparable to other ADMs, within the limitations of the small cohort size. DermACELL’s advanced processing may also reduce complications that lead to reconstruction failure. Histologic analysis reveals early integration at 6 weeks post-implantation.
